# Mental health following an initial period of COVID-19 restrictions: findings from a cross-sectional survey in the Republic of Ireland

**DOI:** 10.12688/hrbopenres.13400.1

**Published:** 2021-12-16

**Authors:** Maria Isabela Troya, Mary Joyce, Ali Khashan, Claire Buckley, Kasturi Chakraborti, Philipp Hoevel, Rory Humphries, Patricia M. Kearney, Elizabeth Kiely, Mike Murphy, Ivan Perry, Ella Arensman

**Affiliations:** 1 School of Public Health, University College Cork, Cork, Ireland; 2 National Suicide Research Foundation, Cork, Ireland; 3 School of Mathematical Sciences, University College Cork, Cork, Ireland; 4 School of Applied Social Studies, University College Cork, Cork, Ireland; 5 School of Applied Psychology, University College Cork, Cork, Ireland; 6 Australian Institute for Suicide Research and Prevention, Griffith University, Brisbane, Australia

**Keywords:** Virus diseases; COVID-19; public health; public mental health; epidemiology; mental health

## Abstract

**Background**: We assessed the mental health of individuals in the general population, during an initial period of easing of COVID-19 restrictions in the Republic of Ireland (RoI).

**Methods:** Data were collected through a nationally representative cross-sectional telephone survey, during the first period of easing of restrictions during the COVID-19 pandemic between May and July 2020. Mental health was examined using the Patient Health Questionnaire Anxiety Depression Scale. Poisson regression analyses were conducted to estimate risk ratios with robust variance estimation of the association between selected demographic factors and the risk of having depression and anxiety symptoms.

**Results**: Of the 1,983 participants, 27.7% (n = 549; 95% CI: 0.26 - 0.30) reported depression and anxiety symptoms, while 74 (3.8%; 95% CI: 0.03 - 0.05) disclosed self-harm and/or suicidal thoughts. Females (RR: 1.60, 95% CI: 1.37 - 1.87, p < 0.0005), employed individuals who experienced a change in work status (RR: 1.50, 95% CI: 1.24 - 1.82, p < 0.0005), participants cocooning due to a health condition (RR: 1.34, 95% CI: 1.08 - 1.66, p< 0.01), participants who were self-isolating (RR: 1.25, 95% CI: 1.03 - 1.51, p=0.025) and moderate-heavy drinkers (RR: 1.27, 95% CI: 1.09 - 1.47, p<0.01) were at increased risk of depression and anxiety. Young people aged 18-29 years and those in the two lowest income categories were most likely to report self-harm and/or suicidal thoughts.

**Conclusion**: As the COVID-19 pandemic continues, with further waves and associated restrictions, the impact on mental health in the population as a whole and in specific subgroups must be considered.

**Study protocol registration**: doi.org/10.12688/hrbopenres.13103.2

## Introduction

Since the appearance of the coronavirus disease in 2019 (COVID-19) and the declaration of a global pandemic by the World Health Organisation in March 2020, individuals and societies have endured ongoing health and social impacts. The implementation of physical distancing interventions has been associated with an overall reduction in COVID-19 incidence worldwide ^1^. Like other Western European countries, the Republic of Ireland (RoI) implemented, with high levels of public compliance, a range of restrictive public health measures in March 2020 to contain the spread of COVID-19 ^2^. These measures included closure of schools and third-level institutes, travel restrictions and physical distancing (see extended data). On the 18 ^th^ May 2020, these restrictions were eased ^3^.

In addition to the physical health impacts and mortality associated with COVID-19, there have been concerns about the effects of physical distancing measures on individuals’ mental health ^4, 5^. Several risk factors for mental health conditions and behaviours including self-harm were identified during the pandemic as a result of social restrictions, including isolation and loneliness, limited access to education and social support, and restricted access to healthcare services ^4– 6^. Emerging evidence indicates there may have been an increase in psychiatric symptoms and self-harm thoughts during the pandemic, particularly among people with pre-existing mental health conditions ^7– 9^. In a study conducted in RoI on 31 ^st^ March 2020 immediately after the announcement of social restrictions, it was found that 20.0 – 22.7% of participants had self-reported symptoms of depression or anxiety ^10^. In a subsequent Irish study in which an online electronic questionnaire and telephone interviews were conducted between 23 ^rd^ April and 1 ^st^ May 2020, 26.6% of respondents reported feelings of loneliness, and 32.5% reported feeling downhearted or depressed ^2^. No study has yet assessed the mental health of individuals in the general population during the period of easing of restrictions in the RoI (May to July 2020).

This study reports on the findings of a national household survey that aimed to assess the mental health of the Irish population during the initial period of easing of COVID-19 related restrictions in the RoI. Specifically, the study objectives were to estimate the prevalence of depression and anxiety symptoms, and thoughts of self-harm and/or suicide based on standardised, validated instruments, and to examine potential associations between selected socio-demographic characteristics and mental health symptomatology.

## Methods

### Ethics

The study received ethical approval from the Clinical Research Ethics Committee of the Cork Teaching Hospitals (Ref: EMC 4 (b)05/05/20). Participants were informed about the research study by the interviewer and verbal consent was obtained before proceeding with the survey. After completion of the survey, participants were advised that further information about the study was available online. Participants were provided with the necessary information to obtain additional information via websites. Respondents could also request these documents by email or post. Further information on ethical considerations and the informed consent process can be found in Troya *et al.* ^11^

As some interview questions had the potential to trigger emotional reactions for participants, training workshops were provided to interviewers in advance of data collection by psychologists from the NSRF and UCC School of Applied Psychology (EA, MM, MIT). At the end of the survey, participants were provided with contact details of support organisations, where indicated. In cases where further follow-up was deemed necessary by the interviewer, phone calls to participants were conducted by the psychology team. In such instances, participants were required to give consent and contact details for a follow-up phone call.

### Study design

This study is part of a larger study which aims to estimate the effects of public health measures in RoI during the COVID-19 pandemic ^11^. The primary outcome was mental health measured through self-reported depression and anxiety symptoms. Secondary outcomes were thoughts of self-harm and/or suicide.

A nationally representative cross-sectional telephone survey was conducted to assess mental health of the Irish population. The survey was conducted during the start of the easing of restrictions. Survey one was administered between 26 ^th^ May - 17 ^th^ June 2020 and survey two between 1 ^st^ – 23 ^rd^ July 2020 ^12, 13^. The response rates were 43.6% for survey one and 26.3% for survey two. Response rates estimates included refusals and calls which were interrupted.

The marketing company Ipsos MRBI conducted the telephone survey in collaboration with the study authors. The study authors designed the survey and provided training to interviewers conducting the telephone survey to ensure a standardised interview and data collection approach.

A patient, who was one of the first cases of COVID-19 diagnosed and managed successfully in RoI contributed to the initial design of this study. The patient contributed to inclusion/exclusion of relevant data sources and measurements ^11^.

### Participants

Participants were randomly selected from the general population. The eligibility criteria were: (a) aged 18 years and above, (b) residing in RoI, (c) having a landline or mobile telephone number, and (d) providing consent. To achieve a nationally representative sample, surveys were conducted using random digit-dialling (approximately 80% mobile, 20% landline), with response rates estimates based on proportion of non-operational and non-answering numbers. A sample size of 1,000 participants, excluding non-responders, non-operational numbers and non-answering numbers, produced a two-sided 95% confidence interval (CI) with a width equal to 0.028 when the sample proportion is 0.05. We aimed to collect data on 1,000 participants during each of two survey iterations. Data were weighted by age, gender, and region, with population estimates based on the Irish Labour Force Survey ^14^.

### Data sources and measurement

Internationally validated instruments were used to measure primary and secondary outcomes. Symptoms of depression and anxiety in the previous two weeks were measured with the 16-item Patient Health Questionnaire Anxiety-Depression Scale (PHQ-ADS) ^15^. The PHQ-ADS is comprised of the PHQ-9 and GAD-7 which measure depression and anxiety, respectively. Scores range from 0 to 48 and participants symptoms are categorised based on their scores. Participants who had scores categorised as mild, moderate, or severe symptoms of depression and anxiety measured by the PHQ-ADS (cut-off >10) were classified as experiencing mental health symptoms. Participants who reported a score of <10 were classified as not experiencing mental health symptoms. The internal reliability of the PHQ-ADS was high (Cronbach’s alpha=0.8 to 0.9) ^15^. Within the PHQ-ADS, one item specifically assesses participants’ self-harm and/or suicidal thoughts in the previous two weeks.

As part of the wider survey, information on socio-demographic characteristics of participants was collected as well as questions about participants’ general health. Participants were also asked questions about cocooning during the pandemic. Cocooning was introduced for people who were advised to stay at home due to increased vulnerability. Measures at the time of this study involved staying at home and avoiding physical contact with others, limiting social interactions and staying within 2km radius of one’s home. Individuals aged 70 or more, as well as individuals who were at higher risk from COVID-19 including those with specific health conditions such as lung conditions, heart disease etc. were advised to cocoon ^16^. A full list of survey questions can be found in Troya *et al*. ^11^. 

Relevant socio-demographic variables included in this study were gender, age group, level of education, net annual income, employment status, children under 18-years-old living in household, cocooning, and alcohol intake.

### Statistical analyses

Statistical software package Stata ^17^ version 15.1 was used to assist in the data analysis. Descriptive statistics summarised selected socio-demographic characteristics, as well as proportions with and without (a) depression and anxiety symptoms, and (b) self-harm and/or suicidal thoughts. Socio-demographic factors were analysed using Chi-square test for categorical variables. Poisson regression analyses were conducted to estimate risk ratios and 95% CIs with robust variance estimation of the association between selected demographic factors (gender, age, change in employment status, household combined annual income, children under 18-years-old living in the household, cocooning, self-reported alcohol intake) and the risk of having depression and anxiety symptoms as measured by the PHQ-ADS. Because we expected the outcome measure to be common (>10%), we wanted to estimate the risk ratio, however, considering that log-linear binomial models often do not converge, which is a known problem with this model, we used Poisson models instead ^18^. Poisson models were performed for depression and anxiety scores, and then for self-harm and/or suicidal thoughts, including all the socio-demographic variables. Given self-harm and/or suicidal thoughts was a rare outcome (n=74), we used the largest number of participants as a reference group: 50 to 69-year-olds and earners of €30,000 - 79,999. Survey commands were used and estimates were weighted to account for the survey sampling design. We repeated the Poisson models for each survey iteration separately to examine whether the results changed between May and July 2020. The significance levels were set at *p* < 0.05.

## Results

A total of 1,983 participants took part in the survey during two iterations of data collected between May and July 2020 (survey one: n = 969; survey two: n = 1,014) ^12^. Socio-demographic characteristics of participants are summarised in Table 1. There were a similar number of males and females (52.0% females, 95% CI: 0.50 - 0.54, n = 1,031) with participants ranging in age from 18-91 years, and a mean age of 47.28 (SD = 17.11). Over half of the sample resided in the region of Leinster, had completed third-level education, and were working as an employee.

**Table 1.** Socio-demographic characteristics of participants.

	Survey 1	Survey 2	Survey 1 & 2
	Frequency (%)	Frequency (%)	Frequency (%)
	*N* = 969	*N* = 1,014	*N* = 1,983
**Gender**			
Male	466 (48.1)	482 (47.5)	948 (47.8)
Female	501 (51.7)	530 (52.3)	1,031 (52.0)
Other	2 (0.2%)	2 (0.2)	4 (0.2)
**Age group**			
18–29 years	167 (17.2)	198 (19.5)	365 (18.4)
30–39 years	167 (17.2)	160 (15.8)	327 (16.7)
40–49 years	182 (18.8)	230 (22.7)	412 (20.8)
50–59 years	157 (16.2)	150 (14.8)	307 (15.5)
60–69 years	157 (16.2)	146 (14.4)	303 (15.3)
70+ years	127 (13.1)	118 (11.6)	245 (12.4)
**Area of residence**			
Leinster	547 (56.4)	585 (57.7)	1,132 (57.1)
Munster	266 (27.5)	247 (24.4)	513 (25.9)
Connacht	104 (10.7)	126 (12.4)	230 (11.6)
Ulster	49 (5.1)	55 (5.4)	104 (5.2)
**Highest level of education**			
Primary level	41 (4.2)	39 (3.8)	80 (4.0)
Group/ Inter/ Junior certificate	76 (7.8)	76 (7.5)	152 (7.7)
Leaving certificate	188 (19.4)	225 (22.2)	413 (20.8)
Other second level/PLC certificate or similar	92 (9.5)	89 (8.8)	181 (9.1)
Third level degree/postgraduate course	566 (58.4)	579 (57.1)	1,145 (57.7)
Other/ don't know	6 (0.6)	6 (0.6)	12 (0.6)
**Household situation**			
Living alone	151 (15.6)	142 (14.0)	293 (14.8)
2 or more sharing (not a couple)	121 (12.5)	105 (10.4)	226 (11.4)
Couple with dependent children	267 (27.6)	310 (30.6)	577 (29.1)
Couple with independent children (no dependent child)	164 (16.9)	167 (16.5)	331 (16.7)
Couple with no children	156 (16.1)	168 (16.6)	324 (16.3)
Lone parent with dependent children	31 (3.2)	33 (3.3)	64 (3.2)
Lone parent with independent children (and no dependent child)	20 (2.1)	26 (2.6)	46 (2.3)
Household with two or more family units living together	57 (5.9)	58 (5.7)	115 (5.8)
Other/ don’t know	2 (0.2)	5 (0.5)	7 (0.4)
**Work situation**			
Working as employee (full-time or part-time)	500 (51.6)	529 (52.2)	1029 (51.9)
Self-employed	90 (9.3)	100 (9.9)	190 (9.6)
Unemployed/ seeking work	81 (8.4)	88 (8.7)	169 (8.5)
Not working due to permanent illness/ disability	41 (4.2)	17 (1.7)	58 (2.9)
Retired	186 (19.2)	194 (19.1)	380 (19.2)
Full-time homemaker/ looking after family	29 (3.0)	32 (3.2)	61 (3.1)
Student	34 (3.5)	51 (5.0)	85 (4.3)
Other/don't know	8 (0.8)	3 (0.3)	11 (0.6)
**Annual combined net income for household**			
Under €19,999	115 (11.9)	111 (10.9)	226 (11.4)
€20,000 to €29,999	109 (11.2)	91 (9.0)	200 (10.1)
€30,000 to €49,999	227 (23.4)	193 (19.0)	420 (21.2)
€50,000 to €79,999	206 (21.3%)	230 (22.7%)	436 (22.0)
€80,000 or greater	128 (13.2%)	152 (15.0%)	280 (14.1)
Don’t know/ refused to answer	184 (19.0%)	237 (23.4%)	421 (21.2)

### Health related information

In terms of alcohol consumption, half of the sample (n = 992; 50.0%, 95% CI: 0.48 - 0.52) described themselves as occasional drinkers, while just over a quarter (n = 513; 25.9%, 95% CI: 0.24 - 0.28) classified themselves as ‘moderate’ drinkers and 32 (1.6%, 95% CI: 0.01 - 0.02) as ‘heavy’ drinkers. Close to one fifth of participants reported cocooning (i.e. avoiding contact with people to avoid risk of COVID-19) (n = 365; 18.4%, 95% CI: 0.16 - 0.19) with the most common reported reason being over 70 years old (n = 214; 10.8%, 95% CI: 0.09 - 0.12). For participants who cocooned because of a health condition (n = 151; 7.6%, 95% CI: 0.07 - 0.09), a severe respiratory condition was the most frequently cited condition (n = 50, 33.1%, 95% CI: 0.07 - 0.11).

### Missing data

There were missing data for the PHQ-ADS for 22 (2.3%) participants in survey one, and for 19 (1.9%) participants in survey two. For the question on suicidal/self-harm thoughts, there were missing data for four (0.4%) participants in survey one, and two (0.3%) participants in survey two.

### Mental health

***Depression and anxiety symptoms.*** More than a quarter of participants (27.7%, 95% CI: 0.26 - 0.30, n = 549) reported symptoms of depression and anxiety as measured by the PHQ-ADS. Of the 549 participants who reported symptoms of depression and anxiety, 383 (69.8%, 95% CI: 0.18 - 0.22) reported mild symptoms, 118 (21.5%, 95% CI: 0.05 - 0.07) reported moderate symptoms and 48 (8.7%, 95% CI: 0.02 - 0.03) reported severe symptoms. The prevalence of depression and anxiety symptoms was similar for survey one (n = 270, 28.5%; 95% CI: 0.26 - 0.32) and survey two (n = 279, 28%; 95% CI: 0.25 - 0.31). Table 2 presents an overview of the number of participants in each PHQ-ADS category as well as a breakdown according to relevant socio-demographic variables.

**Table 2.** Binary classification of PHQ-ADS scores by socio-demographic characteristics for all participants.

	Minimal (PHQ-ADS < 10)	Mild or more (PHQ-ADS ≥ 10)
**Gender**	(n = 1,393)	(n = 549)
Male	733 (52.6%)	194 (35.3%)
Female	659 (47.3%)	353 (64.3%)
Other	1 (0.1%)	2 (0.4%)
**Age group**	(n=1378)	(n=541)
18–29 years	201 (14.6%)	159 (29.4%)
30–39 years	213 (15.5%)	108 (20.0%)
40–49 years	293 (21.3%)	109 (20.1%)
50–59 years	237 (17.2%)	66 (12.2%)
60–69 years	242 (17.6%)	53 (9.8%)
70+ years	192 (13.9%)	46 (8.5%)
**Highest level of education**	(n=1393)	(n=549)
Primary level	52 (3.7%)	21 (3.8%)
Group/ inter/ junior certificate	107 (7.7%)	37 (6.7%)
Leaving certificate	280 (20.1%)	128 (23.3%)
Other second level/PLC certificate or similar	132 (9.5%)	45 (8.2%)
Third level degree/postgraduate course	814 (58.4%)	316 (57.6%)
Other	8 (0.6%)	2 (0.4%)
**Annual combined net income for household**	(n=1393)	(n=549)
Under €19,999	146 (10.5%)	73 (13.3%)
€20,000 to €29,999	136 (9.8%)	58 (10.6%)
€30,000 to €49,999	305 (21.9%)	111 (20.2%)
€50,000 to €79,999	318 (22.8%)	111 (20.2%)
€80,000 or greater	217 (15.6%)	60 (10.9%)
Don’t know/ refused to answer	271 (19.5%)	136 (24.8%)
**Change in employment**	(n=1393)	(n=549)
No change	520 (37.3%)	136 (24.8%)
Change	370 (26.6%)	170 (31.0%)
Not applicable	503 (36.1%)	243 (44.3%)
**Children <18 in household**	(n=1393)	(n=549)
No	963 (69.1%)	352 (64.1%)
Yes	430 (30.9%)	197 (35.9%)
**Cocooning**	(n=1393)	(n=549)
Not cocooning	1007 (72.3%)	376 (68.5%)
Over 70 years old	163 (11.6%)	51 (8.8%)
Health condition	96 (6.9%)	55 (9.5%)
Self-isolating	126 (9.0%)	75 (13.0%)
**Alcohol intake**	(n=1393)	(n=549)
None/ occasional drinker	1015 (72.9%)	389 (70.9%)
Moderate/ heavy drinker	378 (27.1%)	160 (29.1%)

Findings from the Poisson regression analysis indicated that there was a significantly higher risk for females reporting symptoms of depression and anxiety than males, RR: 1.60 (1.37 - 1.86) (see Table 3). Individuals who had previously been employed or self-employed and had experienced a change in their work status had a significantly higher risk for symptoms of depression and anxiety than those for whom there was no change, RR: 1.50 (1.23 - 1.82). There was a significantly higher risk for symptoms of depression and anxiety for participants who were cocooning because of a health condition, RR: 1.33 (1.07 - 1.65), and participants who were self-isolating, RR: 1.24 (1.02 - 1.50). Lastly, participants who classified themselves as ‘moderate’ or ‘heavy’ drinkers were at a significant higher risk for mild or more severe symptoms of depression and anxiety, RR: 1.26 (1.08 - 1.47).

**Table 3.** Risk (RR) for having ‘mild or greater’ symptoms of depression and anxiety as per the PHQ-ADS.

	Crude		Adjusted	
Predictor variable	RR (95% CI)	P value	RR (95% CI)	P value
**Gender**				
Male	Reference [1.00]		Reference [1.00]	
Female	2.023 (1.64, 2.48)	<0.0001	1.60 (1.37, 1.86)	<0.0001
**Age**				
18–29 years	Reference [1.00]		Reference [1.00]	
30–39 years	0.640 (0.46, 0.87)	0.005	0.89 (0.73, 1.09)	0.267
40–49 years	0.470 (0.34, 0.63)	<0.0001	0.69 (0.56, 0.85)	0.001
50–59 years	0.352 (0.24, 0.49)	<0.0001	0.50 (0.39, 0.64)	<0.0001
60–69 years	0.276 (0.19, 0.39)	<0.0001	0.32 (0.24, 0.43)	<0.0001
70+ years	0.302 (0.20, 0.44)	<0.0001	0.24 (0.14, 0.40)	<0.0001
**Education**				
Primary level	Reference [1.00]		Reference [1.00]	
Junior certificate	0.856 (0.45, 1.60)	0.629	0.70 (0.43, 1.15)	0.168
Leaving certificate	1.131 (0.65, 1.95)	0.658	0.72 (0.46, 1.12)	0.156
Other secondary level	0.844 (0.45, 1.55)	0.586	0.58 (0.36, 0.94)	0.029
Third level/PG course	0.961 (0.56, 1.62)	0.882	0.67 (0.43, 1.05)	0.085
Don’t know/ refused	0.619 (0.12, 3.16)	0.564	0.58 (0.15, 2.16)	0.425
**Income**				
Under €19,999	Reference [1.00]		Reference [1.00]	
€20,000 – €29,000	0.852 (0.56, 1.29)	0.454	1.01 (0.76, 1.33)	0.934
€30,000 – €49,000	0.727 (0.51, 1.03)	0.08	0.87 (0.67, 1.13)	0.318
€50,000 – €79,000	0.698 (0.48, 0.99)	0.047	0.82 (0.63, 1.08)	0.171
€80,000 or greater	0.552 (0.37, 0.82)	0.004	0.66 (0.48, 0.91)	0.011
Don’t know/ refused	1.003 (0.70, 1.42)	0.983	0.89 (0.70, 1.14)	0.386
**Change in employment (for those employed)**				
No change	Reference [1.00]		Reference [1.00]	
Change	1.756 (1.35, 2.28)	<0.0001	1.50 (1.23, 1.82)	<0.0001
Not applicable	1.847 (1.44, 2.35)	<0.0001	1.68 (1.37, 2.05)	<0.0001
**Children <18 in household**				
No	Reference [1.00]		Reference [1.00]	
Yes	1.253 (1.01, 1.54)	0.033	1.03 (0.88, 1.20)	0.69
**Cocooning**				
Not cocooning	Reference [1.00]		Reference [1.00]	
Over 70 years old	0.777 (0.54, 1.09)	0.153	1.57 (0.97, 2.56)	0.064
Health condition	1.506 (1.05, 2.14)	0.023	1.33 (1.07, 1.65)	0.009
Self-isolating	1.521 (1.11, 2.08)	0.009	1.24 (1.02, 1.50)	0.025
**Alcohol intake**				
None/occasional drinker	Reference [1.00]		Reference [1.00]	
Moderate/heavy drinker	1.104 (0.88, 1.37)	0.373	1.26 (1.08, 1.47)	0.002

***Suicidal or self-harm thoughts.*** When asked about suicidal or self-harm thoughts in the previous two weeks, 3.8% of the participants (n = 74, 95% CI: 0.03 - 0.05) reported that they had experienced these thoughts on at least a few days during this timeframe. In survey one, 3.3% (n = 32; 95% CI: 0.02 - 0.05) participants reported thoughts of self-harm and/or suicide in the past two weeks, while this number had increased to 4.2% in survey two (n = 42; 95% CI: 0.03 - 0.06). Individuals aged 18 – 29 years were more likely to have suicidal and/or self-harm thoughts as opposed to 50 – 69-year-olds (RR: 3.41; 95% CI: 1.86 - 6.22) (see Table 4). Participants in the two lowest income categories (<€19,999 and €20,000–29,999) were more likely to have suicidal and/or self-harm thoughts than those earning €30,000–79,999 (RR: 2.84; 1.33 - 6.03, and RR: 2.22; 1.03 - 4.80).

**Table 4.** Risk (RR) for having self-harm and/or suicidal ideation in the past two weeks.

		Crude		Adjusted	
Predictor variable	Number of cases	RR (95% CI)	P value	RR (95% CI)	P value
**Gender**					
Male	30	Reference [1.00]		Reference [1.00]	
Female	42	1.298 (0.805, 2.091)	0.284	1.321 (0.84, 2.05)	0.216
**Age**					
18–29 years	32	3.346 (1.830, 6.118)	<0.0001	3.405 (1.86, 6.22)	<0.0001
30–39 years	8	0.876 (0.374, 2.052)	0.761	1.410 (0.57, 3.45)	0.451
40–49 years	10	0.873 (0.396, 1.926)	0.736	1.495 (0.64, 3.47)	0.349
50–69 years	17	Reference [1.00]		Reference [1.00]	
70+ years	4	0.580 (0.193, 1.743)	0.332	0.517 (0.14, 1.84)	0.31
**Education**					
Secondary level or less	39	Reference [1.00]		Reference [1.00]	
Third level/PG course	35	0.636 (0.399, 1.013)	0.057	0.796 (0.47, 1.32)	0.38
**Income**					
Under €19,999	14	2.78 (1.381, 5.596)	0.004	2.844 (1.33, 6.03)	0.007
€20,000 – €29,000	9	1.965 (0.881, 4.383)	0.099	2.223 (1.03, 4.79)	0.042
€30,000 – €79,000	20	Reference [1.00]		Reference [1.00]	
€80,000 or greater	5	0.761 (0.283, 2.047)	0.588	0.842 (0.32, 2.22)	0.729
Don’t know	26	2.759 (0.015, 0.037)	0.001	2.174 (1.20, 3.91)	0.01
**Change in employment (for those employed)**					
No change	17	Reference [1.00]		Reference [1.00]	
Change	22	1.597 (0.839, 3.038)	0.154	1.305 (0.68, 2.47)	0.414
Not applicable	35	1.838 (1.020, 3.312)	0.043	1.066 (0.56, 2.01)	0.843
**Children <18 in house**					
No	56	Reference [1.00]		Reference [1.00]	
Yes	18	0.669 (0.390, 1.148)	0.145	0.697 (0.38, 1.25)	0.232
**Cocooning/self-isolating**					
No	50	Reference [1.00]		Reference [1.00]	
Yes	24	1.210 (0.736, 1.989)	0.452	1.302 (0.76, 2.23)	0.336
**Alcohol intake**					
None/occasional drinker	59	Reference [1.00]		Reference [1.00]	
Moderate/heavy drinker	15	0.662 (0.372, 1.176)	0.16	0.887 (0.50, 1.55)	0.679

## Discussion and conclusions

This research is amongst the first nationally representative studies to report on mental health during a period of easing of COVID-19 related restrictions. Whilst research into on COVID-19 and its long-term impacts continue, this study adds to the data on self-reported mental health outcomes of a nationally representative population in RoI. Over a quarter of participants (27.7%) reported symptoms of depression and anxiety. Females, young people aged 18–29, those who experienced a change in their work situation, moderate to heavy drinkers, individuals cocooning due to health conditions and self-isolating, and those who report moderate to heavy drinking, were at increased risk of adverse mental health outcomes. Young people aged 18–29 and low-income earners (<€29,999) had a higher risk of experiencing self-harm or suicidal thoughts.

In RoI, findings from the Healthy Ireland 2018 survey, a national representative survey, reported rates of 6% of self-reported depression and anxiety before the COVID-19 pandemic ^19^. During the first week of the implementation of movement restrictions measures (31 ^st^ March 2020) in RoI, Hyland *et al*. found 20 - 22.7% of participants had self-reported symptoms of depression or anxiety ^10^. In that early study, females and young people aged 18-34 had higher levels of mental health symptoms ^10^. The findings from the current study, which used the same measurement instrument (PHQ-ADS), and was conducted between May and July 2020, suggest that levels of anxiety and depression may have increased during the period of intense public health restrictions. However, it is worth considering that the overall impact of COVID-19 may also have had an impact on individuals’ depression and anxiety symptoms. Females and young people have also been found to be at increased risk for mental health symptoms in other countries including the United Kingdom ^10, 20^, Germany ^21^ and Ecuador ^22^.

Findings suggest that individuals who were cocooning due to a health condition had increased risk of mental health symptoms, as well as those who were self-isolating for other reasons. Being over the age of 70 was not associated with poorer mental health outcomes. While participants over the age of 70 who were cocooning reported poorer mental health outcomes (RR: 1.57), these findings were not statistically significant (p = 0.064). In other countries, including the Netherlands and United States, increased mental health symptoms were reported in this age group, including an increase in feelings of loneliness ^23, 24^. As the COVID-19 pandemic continues, government advice for older people to self-isolate requires careful calibration given the potential long-term effects on older people’s mental health, including risk of self-harm ^25^.

The RoI implemented public health measures that were broadly similar to those implemented in other Western European countries aligned with WHO recommendations and most recent evidence ^1^. Broadly similar levels of mental health symptoms (anxiety and depression) associated with the pandemic and public health restrictions have been reported in the UK (21.0% - 26.1%) ^20, 26^, Italy (17.0% - 20.0%) ^27^ Germany (14.0% - 44.0%) ^21^, Austria (19.0% - 21.0%) ^28^, and Australia (21.0 - 27.6%) ^29^. Findings from other countries, such as China (28.0% - 35.0%) ^30, 31^ and Bangladesh (33.0% - 57.0%) ^32^, indicate higher levels of anxiety and depression.

This study is among the first to measure the prevalence of self-harm and/or suicidal thoughts in a national general population sample during a period of easing of restrictions of the COVID-19 pandemic. We found that 3.7% of the population reported these thoughts during the initial period of easing of public health restrictions. Another study conducted in the UK with 3,077 participants recruited through an online survey found higher rates of self-harm and/or suicidal thoughts compared to our study and found that they increased over time: survey one (31 ^st^ March to 9 ^th^ April 2020): 8.2%, survey two (10-27 ^th^ April 2020): 9.2%, and survey three (28 ^th^ April to 11 ^th^ May 2020): 9.8% ^20^. Studies conducted prior to the COVID-19 pandemic have found similar rates whereby a meta-analysis of data from multiple European countries reported a 12-month prevalence of 2.9% ^33^. We therefore do not have evidence from the current study that thoughts of self-harm and/or suicide have increased during the first six months of the COVID-19 pandemic, but we should note the relative imprecision with which this rare outcome is estimated in the current study. The findings on socio-demographic factors associated with thoughts of self-harm and/or suicide with increased risk in younger people (aged 18 – 29 years) and low-income earners, are consistent with findings from the National Self-Harm Registry of Ireland ^34^. Thousands of Irish citizens were also in receipt of the COVID-19 unemployment payment due to the closure of businesses which may have led to financial and employment concerns ^35^.

This is the first nationally representative study based on telephone interviews which assesses mental health during the period May to July 2020; a period of easing of COVID-19 related restrictions in RoI. The study reports on individuals’ experiences of the pandemic during the two-month period of easing of restrictions, using standardised tools with high retest-test reliability and internal consistency. As the COVID-19 pandemic continues, and further waves of public health measures are implemented by governments worldwide, there is a clear need for additional and ongoing work, including longitudinal studies on the impact of these measures on individuals’ mental health. Policymakers and clinicians should take note of the groups at higher risk of poor mental health outcomes and target their resources and support accordingly. There is evidence that interventions delivered in primary care and online interventions can aid in the treatment and support of individuals with mental health symptoms associated with the COVID-19 pandemic, including depression, anxiety, and suicidality ^36– 38^.

Given the cross-sectional design, the issue of reverse causation should be considered in the interpretation of the findings ^39^. The response rate for the first survey was relatively high for a population based survey at 43.6%. The rate for the second survey was lower at 26.3%. It is unclear why this disparity occurred between the two waves of data collection which took place within a relatively short timeframe and involved the same team of trained interviewers. One possible explanation is that survey two occurred during a period of further easing of restrictions which may have impeded individuals’ time and availability to respond to a telephone survey. Furthermore, non-participation bias must be considered, given that participation in this study relied on participants answering their telephone and talking about their mental health without prior notice. The study included a wide variety of questions, however, there was certain relevant information that was not recorded such as ethnicity.

In further work, it will be important to assess the potential impact of the pandemic on ethnic minorities, given the evidence of increased morbidity and mortality associated with COVID-19 in these vulnerable groups ^40– 42^. Considering the ongoing impact of the COVID-19 pandemic, future research may wish to follow a nationally representative cohort of participants to examine further mental health impacts of the movement restriction measures of the pandemic. Despite the large sample size in the current study, we examined a relatively rare outcome (self-harm and/or suicidal thoughts) with only 74 observations. Future research with a larger sample size could further examine vulnerable groups at risk of self-harm and/or suicidal thoughts. Lastly, qualitative research could help to explore in further detail the impact of the pandemic on individuals’ mental health and examine strategies to address such impacts.

## Data availability

### Underlying data

Zenodo: COVID-19: Estimating the burden of symptomatic disease in the community and the impact of public health measures on physical, mental and social wellbeing https://doi.org/10.5281/zenodo.5650852 ^12^

This study contains the following underlying data:

- NHS_Survey1.sav
- NHS_Survey2.sav

### Extended data

Harvard Dataverse: Questionnaires for Surveys WP1 and WP2.

https://doi.org/10.7910/DVN/EKUTFF ^13^

This study contains the following extended data:

- Survey 1 questionnaire in DOCX format (Appendix I)
- Survey 2 questionnaire in DOCX format (Appendix II)

This study also contains the following associated data: 

Zenodo: Timeline of public health measures in Ireland during March - July 2020 https://doi.org/10.5281/zenodo.5777656

Data are available under the terms of the Creative Commons Attribution 4.0 International.
