# Supplementary material for: Mental health following an initial period of COVID-19 restrictions: findings from a cross-sectional survey in the Republic of Ireland
Source: HRB Open Res. 2022 Sep 22;4:130. Originally published 2021 Dec 16. [Version 2] doi: 10.12688/hrbopenres.13400.2 (PMC9120930; doi:10.12688/hrbopenres.13400.2)
Supplement: Peer review history

# Peer review: Reviewer response for version 1

**DOI:** 10.21956/hrbopenres.14598.r31874

**Published:** 2022-05-19

**Authors:** Aoife O'Donovan

**Affiliations:** 1 University of California, San Francisco, San Francisco, CA, USA

This study examines mental health in Ireland between May and July 2020, an early phase of the COVID-19 pandemic when Ireland had recently removed strong restrictions on social activities. In this nationally representative study, 27.7% of participants reported clinically significant symptoms of depression and anxiety. The authors compare their results to those obtained in similar studies from March to May 2020 when strict restrictions were in place. In these earlier studies, 20-32% of individuals reported symptoms of depression and/or anxiety. Comparing these data to those collected prior to the pandemic, it appears that levels of depression and anxiety increased with the onset of the COVID-19 pandemic and did not immediately resolve with the official loosening of restrictions.

 One major strength of this study is that it was designed to be nationally representative with participants selected at random from the general population of Ireland. However, due to the relatively modest response rates, the sample is skewed towards a more highly educated and employed sample. Another strength is the use of validated instruments for depression and anxiety (PHQ-9 and GAD-7). One limitation is that the study does not include data on personal efforts to socially isolate, which varied greatly across individuals. Although restrictions were loosened at the time of these surveys, it is possible that many individuals were maintaining restrictions in their own lives. Future surveys should include questions about engagement in protective and risky behaviors and their associations with depression and anxiety.

 The investigators employed a telephone survey to collect all data, with surveys conducted roughly one month apart. Unfortunately, but as is common in such studies, the response rate was modest at 44% for survey 1 and 26% for survey 2. It is likely that there was selective non-participation, such that the current results may underestimate levels of depression and anxiety in the population.

 A small percentage of the sample reported self-harm or suicidal thoughts in survey 1 (n=32) and survey 2 (n=42). The investigators employed the largest group of participants, 50 to 69-year-olds and earners of €30,000-79,999 as reference groups for analyses involving self-harm and/or suicidal thoughts. However, it might have been more informative to simply describe the characteristics of this small group of individuals compared to the rest of the sample. As it stands, the compelling conclusion that young people and low-income earners had higher rates of self-harm/suicidal thoughts only applies to the comparison with middle-aged and middle-income earners. Future studies should pick up on this important issue and identify sociodemographic and economic predictors of self-harm and suicidal thoughts during the pandemic.

 The high socioeconomic status of the sample, as indexed by >50% of the sample, having completed third-level education and the majority being employed, is a limitation because the effects of the pandemic were not felt equally across socioeconomic strata. It would be helpful to conduct additional studies to examine levels of depression and anxiety across the socioeconomic strata, in urban versus rural samples, and as the authors point out, in different ethnic and racial groups.

 Overall, this project is a representation of exactly the kind of work that needs to happen to ensure a data-based approach to managing the mental health effects of infectious disease pandemics. It is interesting that the investigators conducted their work during a relative easing of pandemic-related restrictions. Their findings indicate that the increase in depression/anxiety that occurred with the onset of the pandemic was not resolved immediately with the loosening of restrictions. These important findings need to be further elucidated and tested over time to inform public mental health campaigns.

Is the work clearly and accurately presented and does it cite the current literature?

Yes

If applicable, is the statistical analysis and its interpretation appropriate?

Yes

Are all the source data underlying the results available to ensure full reproducibility?

Yes

Is the study design appropriate and is the work technically sound?

Yes

Are the conclusions drawn adequately supported by the results?

Yes

Are sufficient details of methods and analysis provided to allow replication by others?

Yes

Reviewer Expertise:

Health Psychology, Psychoneuroimmunology

I confirm that I have read this submission and believe that I have an appropriate level of expertise to confirm that it is of an acceptable scientific standard.

# Response

**Published:** 2022-09-02

**Authors:** Mary Joyce

**Affiliations:** National Suicide Research Foundation, Ireland

We thank the reviewer for their time reviewing our manuscript and the constructive feedback provided, as well as highlighting the study’s strengths, limitations, and main findings. We have addressed the reviewer’s comments below:

1. We agree with the reviewer that our study would have benefited from gathering further information on personal efforts to socially isolate as well as engagement in protective and risk behaviours. Future research would benefit from incorporating these questions into their studies. We have now noted this in the Discussion section (see paragraph 8).
2. The reviewer suggests that there may be selective non-participation via the telephone survey and therefore the results may underestimate levels of depression and anxiety in the population. Whilst we acknowledge that our sample may be biased towards people who answer telephone calls, we included both landline (20%) and mobile phone (80%) numbers to obtain the best possible representation. We used the best study design available to us during a pandemic, which did have its limitations, but which aimed to obtain a representative national sample. It could be possible that levels of depression and anxiety in the Irish population are underestimated due to non-participation bias. This had been outlined in the Discussion section and is now further highlighted based on this feedback (paragraph 7).
3. The reviewer suggests that it might have been more informative to describe the characteristics of the small group of individuals who reported self-harm/suicidal thoughts compared to the rest of the sample. To address this point, we have now included an additional table in the Results section to further describe the characteristics of those reporting self-harm/suicidal thoughts in comparison to the full sample (Table 4).
  For this analysis of individuals reporting self-harm or suicidal thoughts, we used the largest number of participants as a reference group: those aged 50-69 years old and €30,000 - 79,999 income earners. We acknowledge the reviewer’s point outlining that these results conclude that young people and low-income earners had higher rates of self-harm/suicidal thoughts only when applied to the comparison with middle-aged and middle-income earners. We have edited the manuscript to reiterate this point in the Discussion (paragraph 1).
4. Our study used random selection to recruit participants, however we did have modest response rates as highlighted by the reviewer. It is suggested by the reviewer that the sample may be skewed towards a more highly educated and employed sample as a consequence. Although we obtained modest response rates, we do not believe this to be the case. Our study sample had the following characteristics: 52% female, 53% aged between 30-59, 57.7% having completed third-level education, and 61.5% employed or self-employed (Troya *et al*., 2021). According to the most recent statistics from the Central Statistics Office (CSO, 2021a), third-level education is completed by 51% of Irish adults as of 2020, and 63.2% were employed in 2020 (CSO, 2021b). Given the similarities in the distribution of gender, age, education, and employment status between our sample and the national demographic profile of adults in Ireland, we believe that our sample could be considered nationally representative. In consideration of any potential skewness in the sample, data were also weighted by age, gender, and region with population estimates based on the Irish Labour Force Survey.
  While we believe that our sample could be considered nationally representative, we agree with the reviewer that the effects of the pandemic were not felt equally across socioeconomic strata. We agree that it would be helpful to conduct additional studies across socioeconomic strata, such as in urban versus rural samples, and have added this to paragraph 8 of the Discussion section.

**References**

 Central Statistics Office. 2021a. Accessed June 21, 2022 from https://www.cso.ie/en/releasesandpublications/ep/p-eda/educationalattainmentthematicreport2020/

 Central Statistics Office. 2021b. Labour Force Survey Quarter 2 2021. Accessed June 21, 2022 from https://www.cso.ie/en/releasesandpublications/ep/p-lfs/labourforcesurveyquarter22021/employment/

# Peer review: Reviewer response for version 1

**DOI:** 10.21956/hrbopenres.14598.r31513

**Published:** 2022-03-01

**Authors:** John R. Kelly

**Affiliations:** 1 Department of Psychiatry, Trinity College Dublin, Trinity College Institute of Neuroscience, Dublin, Ireland; 2 Department of Psychiatry, Tallaght University Hospital, Dublin, Ireland

Troya and colleagues present findings from a nationally representative cross-sectional telephone survey, using the Patient Health Questionnaire Anxiety-Depression Scale, during a period of easing of COVID-19 related restrictions in the Republic of Ireland.

 This is a useful survey and very well written.  

 I have only 3 very minor comments to make.

1. Methods: In paragraph 2, line 3. I recommend adding “National Suicide Research Foundation” (NSRF).
2. Table 1: Why do only 7 boxes have the % sign? Survey 1 column, gender, other, and then in the last 3 rows. Perhaps, there is no need for the % sign.
3. Discussion: In paragraph 1, line 3: “Whilst research into on COVID-19”. No need for on.

In summary, this is a very clear and well-written survey, which adds to the existing research on the mental health impact of COVID-19.

Is the work clearly and accurately presented and does it cite the current literature?

Yes

If applicable, is the statistical analysis and its interpretation appropriate?

Yes

Are all the source data underlying the results available to ensure full reproducibility?

Yes

Is the study design appropriate and is the work technically sound?

Yes

Are the conclusions drawn adequately supported by the results?

Yes

Are sufficient details of methods and analysis provided to allow replication by others?

Yes

Reviewer Expertise:

Psychiatry

I confirm that I have read this submission and believe that I have an appropriate level of expertise to confirm that it is of an acceptable scientific standard.

# Response

**Published:** 2022-09-02

**Authors:** Mary Joyce

**Affiliations:** National Suicide Research Foundation, Ireland

We thank the reviewer for their time reviewing our manuscript and the helpful feedback provided. We have addressed the reviewer’s comments and incorporated them into our manuscript as evidenced below.  

1. We have added the full acronym of the NSRF (National Suicide Research Foundation) acronym to paragraph 2 of the Methods section.  
2. We have amended Table 1 to address the typing error whereby the % sign was included in seven cells in error.  
3. We have removed the word “on” from the first paragraph of the Discussion section. 
